# Dietary supplementation with a specific combination of high protein, leucine, and fish oil improves muscle function and daily activity in tumour-bearing cachectic mice

**DOI:** 10.1038/sj.bjc.6604905

**Published:** 2009-03-03

**Authors:** K van Norren, D Kegler, J M Argilés, Y Luiking, M Gorselink, A Laviano, K Arts, J Faber, H Jansen, E M van der Beek, A van Helvoort

**Affiliations:** 1 Nutrition and Pharmacology Group, Division of Human Nutrition, Wageningen University, The Netherlands; 2 Cancer Research Group, Departament de Bioquímica i Biologia Molecular, Facultat de Biologia, Universitat de Barcelona, Spain; 3 Center for Innovative Consumer Studies, Wageningen UR,, The Netherlands; 4 Department of Clinical Medicine, University La Sapienza, Rome, Italy; 5 Department of Food and Pharmacology, University of Wageningen, The Netherlands

**Keywords:** muscle, wasting, nutrition, cachexia, leucine, fish oil

## Abstract

Cancer cachexia is characterised by metabolic alterations leading to loss of adipose tissue and lean body mass and directly compromises physical performance and the quality of life of cancer patients. In a murine cancer cachectic model, the effects of dietary supplementation with a specific combination of high protein, leucine and fish oil on weight loss, muscle function and physical activity were investigated. Male CD2F1 mice, 6–7 weeks old, were divided into body weight-matched groups: (1) control, (2) tumour-bearing, and (3) tumour-bearing receiving experimental diets. Tumours were induced by s.c. inoculation with murine colon adenocarcinoma (C26) cells. Food intake, body mass, tumour size and 24 h-activity were monitored. Then, 20 days after tumour/vehicle inoculation, the animals were killed and muscle function was tested *ex vivo*. Tumour-bearing mice showed reduced carcass, muscle and fat mass compared with controls. EDL muscle performance and total daily activity were impaired in the tumour-bearing mice. Addition of single nutrients resulted in no or modest effects. However, supplementation of the diet with the all-in combination of high protein, leucine and fish oil significantly reduced loss of carcass, muscle and fat mass (loss in mass 45, 52 and 65% of TB-con, respectively (*P*<0.02)) and improved muscle performance (loss of max force reduced to 55–64% of TB-con (*P*<0.05)). Moreover, total daily activity normalised after intervention with the specific nutritional combination (50% of the reduction in activity of TB-con (*P*<0.05)). In conclusion, a nutritional combination of high protein, leucine and fish oil reduced cachectic symptoms and improved functional performance in cancer cachectic mice. Comparison of the nutritional combination with its individual modules revealed additive effects of the single components provided.

Cancer cachexia is one of the most debilitating aspects of cancer and has been associated with increased morbidity and mortality, reduced quality of life, impaired response to chemotherapy, increased susceptibility to chemotherapy-induced toxicity and a higher incidence of post-operative complications (Argiles, 2005). A consensus on the definition for cachexia has been reached at the 4th International Conference on Cachexia at Tampa (Consensus definition cachexia/Cachexia Conference Tampa 2007: ‘Cachexia, also known as Wasting Disease, is a complex metabolic syndrome associated with underlying illness and characterized by loss of muscle with or without loss of fat mass. The prominent clinical feature of cachexia is weight loss in adults (corrected for fluid retention) or growth failure in children (excluding endocrine disorders). Anorexia, inflammation, insulin resistance and increased muscle protein breakdown are frequently associated with cachexia. Cachexia is distinct from starvation, age-related loss of muscle mass, primary depression, malabsorption and hyperthyroidism and is associated with increased morbidity’.) (Evans *et al*, 2008). Cancer cachexia is characterised by involuntary weight loss with a depletion of not only fat mass, but also lean body mass due to muscle wasting. Symptoms besides weight loss are debilitation, weakness, oedema, impaired immune response, and a decline of motor and mental functions (Argiles, 2005). Approximately 45% of cancer patients loose more than 10% of their pre-diagnostic weight (Argiles, 2005). The tumour can induce changes in protein metabolism, resembling those found in infection or injury (Argiles, 2005). These changes are characterised by a net protein breakdown and an increased oxidation of branched-chain amino acids (BCAAs), especially in muscle, to support energy supply (e.g., gluconeogenesis from amino acid carbon skeletons) and to provide amino acids for the synthesis of acute phase proteins ((Baracos and Mackenzie, 2006; Choudry *et al*, 2006) for reviews). The breakdown of the host protein is partly stimulated by inflammatory mediators (e.g., TNF*α*, IL-6) produced not only by the host, but also by the tumour (Argiles, 2005).

We hypothesise that nutritional support in cancer patients should aim for a direct counteraction of the net body protein breakdown, rather than for a mere increase in caloric intake. To establish a net positive protein balance, protein supplementation should be combined with specific components modifying and mitigating both the catabolic and anabolic signals.

Fish oil derived N-3 polyunsaturated fatty acids (PUFAs) (Colomer *et al*, 2007) may have anti-catabolic effects through a reduction of the inflammatory state. The vast majority of the clinical trials in which fish oil derived n-3 PUFAs were tested report an increase (Wigmore *et al*, 1996, 2000; Barber *et al*, 1999, 2001; Jatoi *et al*, 2004) or maintenance (Burns *et al*, 1999, 2004; Fearon *et al*, 2003) of body weight (BW), whereas in two other clinical trials no effect on the loss of BW was found (Gogos *et al*, 1998; Bruera *et al*, 2003). In the latter, however, the supplementation period was only 2 weeks (Bruera *et al*, 2003) and/or only a small number of patients was included (Gogos *et al*, 1998). In a clinical trial in pancreatic cancer patients, in which a fish oil-enriched product was tested against an isocaloric/isonitrogenous placebo, no differences between groups were found on either LBM or physical activity. However, when compared with baseline values, total energy expenditure, resting energy expenditure and physical activity level increased significantly in those randomised to the n-3 enriched supplement (Moses *et al*, 2004). Other studies on supplementation with EPA or fish oil, specifically in cancer patients, showed a net lean tissue mass gain (Fearon *et al*, 2003), an increase in total resting energy expenditure and physical activity, a decreased need for total parenteral nutrition (TPN) (Kenler *et al*, 1996) and an improved quality of life (Fearon *et al*, 2003; Burns *et al*, 2004). One study suggested improved survival (Gogos *et al*, 1998).

Protein synthesis cannot occur without the presence of the building units, the amino acids. In fact, a high supply of essential amino acids has been described to be essential for increasing protein synthesis (Rooyackers and Nair, 1997), indicating that not only the quantity, but also the quality of the amino acid supply is vital. Therefore, the protein fraction supplemented has to be rich in essential amino acids.

BCAAs and especially leucine are known to control skeletal muscle protein metabolism (Kobayashi *et al*, 2006) by stimulating protein synthesis and inhibiting protein breakdown. Prospective caloric- and nitrogen-controlled trials of BCAA supplementation through TPN) in septic patients indeed resulted in an improvement of pre-albumin levels and a decreased overall mortality (Jimenez Jimenez *et al*, 1991; Garcia-de-Lorenzo *et al*, 1997). Anthony and co-workers showed in various rat models that leucine enhances protein synthesis in skeletal muscle through both insulin-dependent and -independent mechanisms (Anthony *et al*, 2002a, 2002b). In tumour-bearing rats a diet supplemented with 3% leucine has been reported to reduce loss of lean body mass, gastrocnemius muscle mass and myosin content, when compared to an isonitrogenous and isocaloric control diet (Gomes-Marcondes *et al*, 2003). These data are supported by the observation that leucine increased protein synthesis in pregnant tumour-bearing rats, possibly resulting from changes in the ubiquitin–proteasome system (Ventrucci *et al*, 2004). In humans, leucine supplementation resulted in an improved muscle protein synthesis in young (Koopman *et al*, 2005) and elderly volunteers (Katsanos *et al*, 2006; Koopman *et al*, 2006; Rieu *et al*, 2006). There are two clinical trials in cancer patients that studied oral BCAA supplementation after surgical removal of the tumour. These studies reported a shorter hospital stay, a better performance status at 3 months and an increased body mass at 1 year following surgery (Surgery, 1997; Meng *et al*, 1999). BCAAs have also been supplemented in the presence of the tumour: patients undergoing chemotherapy received oral BCAA supplementation up to 1 year after the start of chemotherapy, resulting in a lower overall morbidity, an improved nutritional status and a better quality of life (Poon *et al*, 2004).

We hypothesise that nutritional support in cancer patients is most effective if aimed simultaneously for a variety of parameters involved in cachexia. Therefore, we studied, in a cancer cachectic murine model, the effect of a combined nutritional supplementation high in protein, rich in leucine and EAA and fish oil compared with single-compound supplementation, on cancer-induced weight loss. Next, also the effects on clinically relevant functional readouts, such as muscle function and physical activity were investigated. To this end the colon 26 adenocarcinoma bearing the cachectic mouse model was used, which has repeatedly shown the loss of muscle mass and muscle function without significant effects on food intake (Tanaka *et al*, 1990; Diffee *et al*, 2002; Gorselink *et al*, 2006), which makes it a suitable model to study the effect of specific nutritional ingredients on cancer cachexia.

## Materials and methods

### Animals

Male CD2F1 mice at 6–7 weeks, (BALB/c × DBA/2, Harlan/Charles River, the Netherlands) were individually housed in a climate-controlled room (12 : 12 dark–light cycle with a constant room temperature of 21±1°C). After acclimatisation for 1 week mice were divided into weight-matched groups: (1) control receiving control chow, (2) tumour-bearing receiving control chow, and (3) tumour-bearing receiving experimental diets. Data shown are derived from the combination of several experimental runs with identical animal characteristics and experimental procedures (unless stated otherwise) and differ only in the experimental diets used. All experimental procedures were approved by the Animal Ethical Committee (DEC consult, Bilthoven, The Netherlands) and complied with the principles of good laboratory animal care.

### Experimental diets (categories A and B experiments)

Experiments are divided in: (A) experiments designed to test the effect of single or combined nutritional components (addition of high protein (hpr), leucine (leu), fish oil (fo)), added to the background diet (AIN93-M) and supplied as pellets; (B) experiments designed to test the effect of a complex nutritional combination that resembles the composition of the new generation FortiCare (Nutricia Advanced Medical Nutrition).

The AIN93-M control diet in the category A experiments contained per kg feed: 126 g protein (100% casein), 727 g carbohydrates and 40 g fat (100% soy oil) (Research Diet Services, Wijk bij Duurstede, the Netherlands). Experimental diets in this category were adjusted to control diets by partly replacing the carbohydrates and/or soy oil by protein and leucine (151 g casein/kg and 16 g leucine/kg feed; TB+hpr+leu), high protein and fish oil (151 g casein/kg and 22 g fish oil/kg feed; TB+fo+hrp), or high protein and leucine and fish oil (151 g casein/kg, 16 g leucine/kg and 22 g fish oil/kg food; TB+fo+hpr+leu). The 22 g of fish oil contained 6.9 g EPA and 3.1 g DHA in a ratio of 2.2 : 1. In summary, in experiment A the following diets and interventions were compared: (1) no tumour, AIN93-M fed (Con); (2) tumour-inoculated AIN93-M fed (TB-con); (3) tumour-inoculated fed AIN93-M with additional protein and leucine (TB+hpr+leu); (4) tumour-inoculated fed AIN93-M with additional fish oil (TB+fo); (5) tumour-inoculated fed AIN93-M with additional fish oil and protein (TB+fo+hrp); (6) tumour-inoculated fed AIN93-M with additional fish oil, high protein and leucine (TB+fo+hpr+leu).

In the category B experiment, the control diet was a more humanised diet, isocaloric and isonitrogenous to the control diet in the A category of experiments, and contained per kg feed 126 g protein (casein), 53 g fat (corn oil), and 699 g carbohydrates. The isocaloric experimental diet (further referred to as Specific Nutritional Composition; SNC) contained per kg feed: 210 g protein (189 g intact protein of which 68% casein and 32% whey and 21 g free leucine), 53 g fat (20.1 g corn oil, 10.2 g canola oil, and 22.2 g fish oil (providing 6.9 g EPA and 3.1 g DHA)), 561 g carbohydrates, 18 g galacto-oligosaccharides and 2 g fructo-oligosaccharides. In experiment B, diets were supplied as dough for product technical reasons.

### Tumour model

Murine C-26 adenocarcinoma cells were cultured *in vitro* with RPMI-1640 supplemented with 5% fetal calf serum and 1% penicillin–streptomycin (Tanaka *et al*, 1990). Tumour cells were trypsinised in a sub-confluent state and, after washing, suspended in Hanks’ balanced salt solution (HBSS) at a concentration of 2.5 × 10^6^ cells ml^−1^. Under general anaesthesia (isoflurane/N_2_O/O_2_), tumour cells (5 × 10^5^ cells in 0.2 ml) were inoculated subcutaneously into the right inguinal flank of the mice. Control (C) animals received a sham injection with 0.2 ml HBSS.

### Experimental protocol

Following inoculation of tumour cells or HBSS, body mass, food intake and tumour size (length and width) were measured three times a week. Only in the category B experiment, daily activity in the home cage was monitored. In all experiments, animals were anaesthetised and weighted at day 20 after tumour inoculation. Skeletal muscles (e.g., m. Tibialis Anterior (mTA), m. Gastrocnemius (mG), m. Extensor Digitorum Longus (mEDL) and M Soleus (mS)), the tumour, spleen, kidneys, liver, epididymal fat, thymus, lungs and heart were dissected and weighed. Carcass mass was calculated by subtracting tumour mass from body mass. In addition, muscle function was tested *ex vivo* in the category B experiment.

### Assessment of daily activity

Physical activity was monitored continuously (24 h) during the 20-day study period starting at day 2, using activity sensors (dual technology detector DUO 240, Visonic; adapted by R Visser, NIN, Amsterdam, The Netherlands) that translated individual changes in the infrared pattern caused by movements of the animals into arbitrary activity counts. Sensors were mounted above the home cages and were connected through input ports and interface to a computer equipped with MED-PC IV software for data collection (MED associates, St Albans, VT, USA). Activity was expressed in counts per hour (both for the total 24-h period, the dark period (active period) and the light period (inactive period)). Activity was calculated for each mouse separately and was expressed relative to its own total activity on day 2, to correct for differences in the individual sensitivity of sensors. The activities of two subsequent days were averaged, to dampen the day to day variability. To determine changes in the activity pattern throughout the experiment, hourly and dark–light activity were expressed as percentage of total daily activity and translated into an actogram.

### Assessment of muscular functionality

Contractile characteristics of the right EDL muscle were assessed *ex vivo*, as described previously (Gorselink *et al*, 2006). Briefly, muscles were allowed to stabilise in the organ bath for 30 min, after which optimal stimulation current and strength were determined. Then force-frequency characteristics (10–167 Hz, 250 ms) were determined and after replenishing the organ buffer and a resting period of 5 min, muscles were subjected to an exercise protocol (83 Hz, 250 ms every 1000 ms). This protocol represents a moderate load, comparable with normal daily activity. At the frequency used, complete tetanus of the muscle is reached. Isometric force signals of the force–frequency curve were analysed for maximal and total force and for maximal contraction and relaxation velocity.

### Statistics

All data are expressed as means±s.e.m. Statistical analyses were performed using SPSS 15.0 (SPSS Benelux, Gorinchem, the Netherlands). In experiment A different batches of animals were used, therefore, for all parameters it was defined that combination of data was allowed if no interaction between groups and experiments were present. Body composition data, tumour and organ masses on day 20 were compared between groups with analysis of variance (ANOVA) and *post hoc* LSD. Differences were considered significant at a *P*-value below *α*/*k*; in which *α*=10% and *k*=amount of comparisons. For experiment A the *P*-value had to be below 0.02; for experiment B the *P*-value had to be below 0.05. Data on food intake, body weight, daily activity, and muscle function that were monitored during the 20 days after inoculation were analysed by repeated measures ANOVA. To further discriminate the differences between groups, the differences or deltas from the first measurement in the range were calculated. These deltas were compared between groups using ANOVA, with *post hoc* LSD for pairwise comparison between groups. For skeletal muscle function, data of first measurement at day 20 were not similar between groups, therefore further discrimination was performed in a per point analysis ANOVA. Differences were considered significant at a two-tailed *P*<0.05.

## Results

### Effects of single *vs* combined nutritional components on different parameters of cachexia

**Figure 1:**
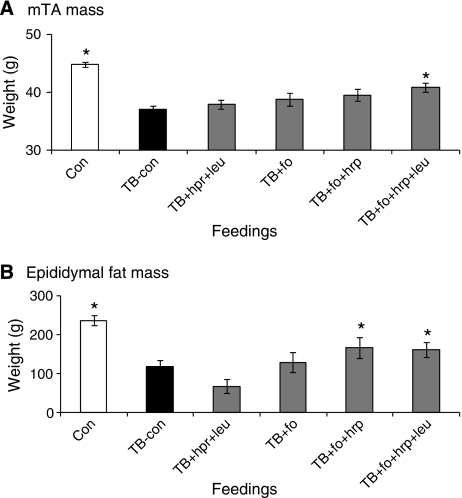
Differences in muscle and fat mass at day 20 after interventions with single or combined nutritional components (Experiment A). (**A**) muscle Tibialis Anterior mass and (**B**) Epididymal fat mass. Con=mice receiving control diet A (AIN93), TB-con=tumour-bearing mice receiving control diet A (AIN93), hpr=high protein, leu=leucine, fo=fish oil. Data are means±s.e.m.; ^*^significantly different from TB-con (*P*<0.02) (*k*=5, *α*=10%) (For more details about statistics, see the Materials and Methods section).

Compared with control mice (Con), carcass and body weight were significantly lower in tumour-bearing control mice (TB-con) on day 20 after tumour inoculation (Table 1A). For all parameters measured there was no group times experiment interaction. The loss of body weight in TB-con mice was derived from both loss of muscle mass and fat mass (e.g., epididymal fat) (Figure 1A and B). No differences in food intake were present between groups for complete curves. When analysed separately per day, at day 20, Con was significantly different from TB-con. None of the tumour-bearing groups were significantly different from each other (Table 1B). Addition of extra protein and leucine (TB+hpr+leu) or fish oil (TB+fo) did not change body weight compared with TB-con (Table 1A). However, addition of fish oil to extra protein (TB+fo+hpr) or fish oil to extra protein and leucine (TB+fo+hpr+leu), resulted in a significant higher fat mass compared with TB-con (Figure 1B). Only the supplementation of the diet with the all-in combination of high protein, leucine and fish oil (TB+fo+hpr+leu) resulted in a significant improvement of body and carcass weight (Table 1A), and of muscle (mTA) and fat (epididymal) mass, compared with TB-con mice (Figure 1). Additive effects of the combination of leucine and high protein were found for muscle mass of the mTA in the presence of fish oil. Addition of each component increased muscle mass stepwise (Figure 1A).

### Effect of a specific nutritional combination on parameters of cachexia

Body and carcass weight were significantly lower in tumour-bearing mice (TB-CON) compared with control mice (CON) on day 20 (Table 2A). The difference in body weight change already being significant at day 15 after tumour inoculation (Table 2C). Again, a significant lower fat mass (epididymal fat) and muscle mass was observed in the TB-CON mice (Table 2B). Food intake was not different between groups (Table 2D). The tumour-bearing mice receiving the Specific Nutritional Combination (TB-SNC group) had a higher body weight, and delta body weight compared with TB-CON mice. The attenuation of body weight loss in the TB-SNC mice coincided with a reduction of fat loss and a reduction in muscle wasting (mTA, mG, and mS) (Table 2B).

Organ (wet) mass of kidney, liver, thymus and heart either decreased with increased cachexia or showed no change (Table 3). Nutritional supplementation resulting in increased carcass weight partly compensated the weight loss. For experiment B the data for organ masses (in percentage of control (CON) ±s.e.m.) were: kidney: TB-CON: 81%±2; TB-SNC: 91%±2, liver TB-CON: 88%±2; TB-SNC: 92%±3, thymus TB-CON: 46%±4; TB-SNC: 55%±4, heart TB-CON: 86%±2; TB-SNC: 88%±2, and lung: TB-CON: 98%±2; TB-SNC: 103%±3. Tumour mass was not increased by any of the nutritional supplementations (Tables 1 and 2).

### *Ex vivo* muscle function (category B experiment)

**Figure 2:**
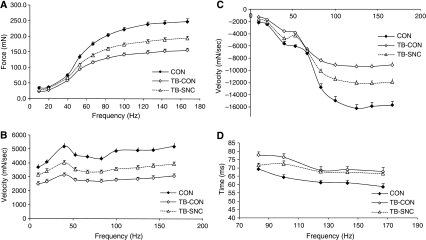
The effect of the specific nutritional combination on skeletal muscle function: force frequency curve (*ex vivo* experiment B). CON=mice receiving control diet B, TB-CON=tumour-bearing mice receiving control diet B, TB-SNC=tumour-bearing mice receiving the specific nutritional combination. Data are means±s.e.m.; data were significantly different from TB-CON when *P*<0.05 (*k*=2, *α*=10%). (**A**) Maximal contraction force (complete curves significantly different from each other *P*<0.01). (**B**) Maximal contraction velocity (complete curves significantly different from each other *P*<0.01). (**C**) Maximal relaxation velocity (complete curves significantly different from each other *P*<0.01). (**D**) CT90: time needed for contraction from 10 to 90% of maximal force (CON significantly different from TB-CON for range 83–176 Hz; TB-SNC significantly different from TB-CON for range 83–100 Hz).

**Figure 3:**
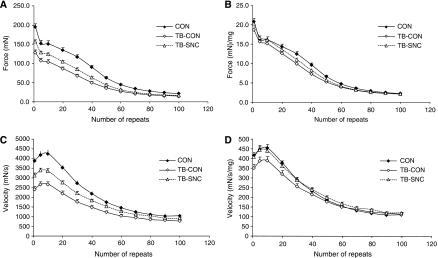
The effect of the specific nutritional combination on skeletal muscle function during exercise (*ex vivo* experiment B). CON=mice receiving control diet B, TB-CON=tumour-bearing mice receiving control diet B, TB-SNC=tumour-bearing receiving the specific nutritional combination. Data as means±s.e.m.; data were significantly different from TB-CON when *P*<0.05, *k*=2, *α*=10%). (**A**) Maximal contraction force (both curves are significantly different from TB-CON till repeat 70). (**B**) Maximal contraction force corrected for muscle mass (CON is significantly different from TB-CON for repeats 30–50; TB-SNC not significant different from TB-CON). (**C**) Maximal contraction velocity (both curves significantly different from TB-CON till repeat 70). (**D**) Maximal contraction velocity corrected for muscle mass (CON significantly different from TB-CON for the first 30 repeats (except for repeat 5 (*P*=0.06)); TB-SNC significantly different from TB-CON for the first 10 repeats).

Force–frequency characteristics (10–167 Hz, 250 ms) were determined *ex vivo* in mEDL. Maximal force, maximal contraction velocity and maximal relaxation velocity were significantly different in TB-CON when compared with CON and TB-SNC (Figure 2A, B and C). When these parameters were corrected for muscle mass, overall curve positions were maintained. Significant differences, however, remained only between CON and TB-CON. To further investigate muscle mass-independent changes in muscle function the time needed for a contraction (CT90) was determined. CT90 was defined as the time needed to go from 10 to 90% of maximal contraction force, at frequencies at which tetanus was obtained. CT90 was significantly different between TB-SNC and TB-CON at lower frequencies at which total tetanus could be obtained (83 and 100 Hz). These data suggest that at frequencies (83–100 Hz) relevant for efficient physical performance (tetanus present), besides muscle mass-dependent changes, also muscle mass-independent changes had occurred that were corrected by specific nutritional intervention. Therefore, an exercise protocol of 100 repeated pulses was applied at 83 Hz. Again, CON and TB-SNC were significantly different from TB-CON during the whole exercise protocol for maximal contraction force (Figure 3A) and maximal contraction velocity (Figure 3C). When maximal contraction force was corrected for muscle mass (Figure 3B) curve positions remained, with only significant differences between CON and TB-CON. Maximal contraction velocity of the TB-SNC group; however, was still significantly different from TB-CON when corrected for muscle mass in the first repeats of the exercise (<10 repeats) (Figure 3D).

### Physical activity (category B experiment)

**Figure 4:**
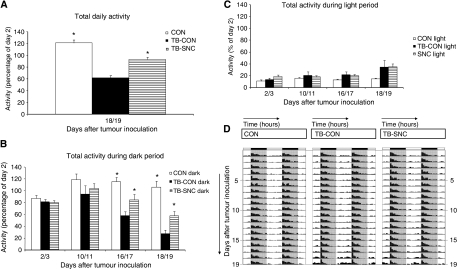
Daily activity (*in vivo* experiment B). (**A**) Total daily activity as % of daily activity on day 2 for all groups. A significant time × group interaction was observed (*P*<0.01). (**B**) Total activity in the dark as percentage of daily activity on day 2 for all groups. (**C**) Total activity in the light as percentage of daily activity on day 2 for all groups. (**A**–**C**): ^*^P<0.05 *vs* TB-CON. (**D**) Actogram, representing percentages of daily activity during the light period from 7–19 h (white shaded areas) and during the dark period from 19–7 h (grey shaded areas) on days 1–19 (vertical) for all groups separately.

Total daily activity showed a significant interaction between time and group (*P*<0.01; RM-ANOVA) over the total period (2–19 days). Activity levels in TB-CON mice were significantly lower than in control mice on days 10–11 (*P*<0.05), and from day 16 onwards (*P*<0.01). The TB-SNC animals did not differ significantly from the control animals in their total activity throughout the experiment, whereas their activity was significantly higher at days 18–19 compared with TB-CON mice (*P*<0.05) (Figure 4A). These differences in total activity resulted from significant changes during their active period (i.e., dark period) (Figure 4B). Throughout the dark period, TB-CON mice were significantly less active than controls on days 16–17 and 18–19 (*P*<0.01), resulting in a drastic decrease in overall activity in the TB-CON mice. The TB-SNC mice were less active than control mice during the dark on day 18–19 (*P*<0.05), but more active than TB-CON mice on those days (*P*<0.05).

Besides a reduction in daily activity level of TB-CON mice, a clear shift in daily activity pattern was observed, that is, from dark to light, both in tumour-bearing controls and in TB-SNC animals on days 18–19 (Figure 4C). To focus on possible shifts in daily activity pattern, hourly activity pattern during the day was expressed as a percentage of the total (100%) daily activity on that specific day (Figure 4D) (i.e., not referring to day 2 and not corrected for the graduate decline in activity for the tumour-bearing groups). At baseline (days 2–3) all groups showed comparable day/night rhythms. Animals were active during the dark and had an inactive period during the light. A relative shift towards increased activity during the light period is observed in the TB-CON group from day 16, which occurs less or delayed in the TB-SNC group.

## Discussion

In this study, the effect of a nutritional supplementation with single *vs* multiple components on body composition in the murine C26 carcinoma model was investigated. In contrast to supplementation of the single ingredients, the combination demonstrated a significant effect on the body composition. In addition, the specific nutritional combination also improved muscle function. Moreover, activity patterns as well as overall daily activity improved, probably as a consequence of improved body composition and muscle function. This clearly supports the added value of a multi target approach, in which catabolism (both protein and fat) is targeted through a reduction of inflammation through the addition of fish oil; whereas anabolism is specifically targeted by protein synthesis through the supplementation of high protein and leucine. The effects on body composition and performance are highly relevant to the clinical situation, because muscle function and daily activity are important contributors to the quality of life of the cancer patient (Morrow *et al*, 2002). Moreover, from a retrospective study in 1555 patients with gastrointestinal carcinomas it was concluded that patients with weight loss had a poorer outcome from treatment than patients without weight loss. This difference contributed to the fact that the patients with weight loss received significantly less chemotherapy and experienced more toxicity from the treatment (Andreyev *et al*, 1998). These findings indicate that maintenance of body composition may beneficially contribute to compliance of the patient to the therapy.

The data focus on the specific needs of the cancer patient and the possible role for nutrition in improving or preventing cachexia characteristics. The data show the effects of different isocaloric nutritional interventions with single ingredients or combinations in the C26 murine model of cancer-induced cachexia. There were no significant differences on food intake between groups on complete curves nor on analysis per day up to day 19. These data confirm earlier findings that for cachexia, validated C26 adenocarcinoma mouse model can be used as a cachectic non-anorectic model (Tanaka *et al*, 1990; Diffee *et al*, 2002; Gorselink *et al*, 2006) The observation, however, that in experiment A, food intake of Con is significantly higher than that of TB mice on day 20 specifically indicates that if tumour growth would continue for a few days more, the tumour-bearing animals would likely become anorectic. The differences in cachectic parameters between control and tumour-bearing mice 20 days after tumour inoculation, were comparable in magnitude to those described in other studies also using the C26 adenocarcinoma mouse model (Lazarus *et al*, 1996; Matsumoto *et al*, 1999; Samuels *et al*, 2000; Fujimoto-Ouchi *et al*, 2006; Gorselink *et al*, 2006).

None of the single components had direct effects. Only the combination of fish oil and high protein increased fat mass. Fat mass has been suggested to be important in survival of the patient (Kalantar-Zadeh *et al*, 2007) whereas muscle mass has been implicated to contribute specifically to the quality of life of the patient (Muscaritoli *et al*, 2004). The data on mTA muscle mass show that at the tested concentration the combination of all components, that is, fish oil, high protein and leucine were needed for a significant effect on muscle mass (Figure 1). Moreover, the results are in line with the hypothesis that next to an increase in anabolic responses, protein catabolism has to be decreased by reduction of inflammation to reach a positive effect on muscle protein mass in a cancer cachectic state. There is growing support that the inflammatory response to a tumour attributes considerably to the progression towards cachexia. It has also been suggested that the increase in catabolic *vs* anabolic processes contributes to the failure to accumulate lean body mass even when nutritional intake is normal (Argiles, 2005). Clinical data from different cachectic patient groups (Wigmore *et al*, 1996, 2000; Barber *et al*, 1999, 2001; Jatoi *et al*, 2004) suggest that fish oil might reduce catabolism and weight loss. Fish oil probably not only attenuates the tumour-induced inflammatory response, but also normalises the insulin resistance present in the cachectic state (Wigmore *et al*, 1996, 2000; Barber *et al*, 1999, 2001; Jatoi *et al*, 2004). Prolongation of survival has been reported in a mixed group of advanced cancer patients supplemented with n-3 fatty acids and vitamin E (Gogos *et al*, 1998) which might also result from immune-modulation. The suggestion that in cachectic patients fish oil might contribute to the maintenance of body composition by a reduction of inflammatory responses, is supported by our data. High protein with leucine (hpr+leu), did not result in significant changes in mTA mass. However, when fish oil was added, the combination of high protein and leucine (fo+hpr+leu) contributed to a significant weight gain of mTA. Therefore, it is hypothesised that reduction of the inflammatory state by fish oil improved the sensitivity of the animals to anabolic stimuli like leucine and high protein, resulting in improved maintenance of muscle protein mass.

Results from *in vivo* studies suggest that BCAAs and especially leucine regulate skeletal muscle protein metabolism (Kobayashi *et al*, 2006). This signal is related to activation of the mTOR pathway (Kimball and Jefferson, 2006). In healthy volunteers, leucine has been reported to provide a signal for stimulation of muscle protein synthesis and to possibly decrease muscle protein breakdown (Rennie *et al*, 2006). In healthy individuals this signal is likely to be short-lived due to the ‘muscle-full phenomenon’ induced by normal nutritional intake and homeostatic control mechanisms (Rennie *et al*, 2006). In contrast, long lasting effects of BCAA supplementation were reported in patients with a metabolic or nutritional deficiency like in septic or cancer patients. In these patient groups BCAA supplementation was reported to result in positive effects on albumin status, quality of life and overall survival (Jimenez Jimenez *et al*, 1991; Garcia-de-Lorenzo *et al*, 1997; Surgery, 1997; Meng *et al*, 1999; Poon *et al*, 2004). Moreover, it has been reported that protein synthesis can only be stimulated in the presence of a high supply of balanced amounts of essential amino acids (Rooyackers and Nair, 1997). Altogether, these studies suggest that a combination of high protein and BCAA supplementation might result in improved protein metabolism, resulting in muscle mass gain, which could contribute to a lower morbidity and a higher quality of life. Our data indeed suggest that both leucine and high protein supplementation contribute to the cumulative effect on muscle mass maintenance, reached by the total nutritional combination (Figure 1 and Table 1).

Combination of supplementation of high protein, leucine and fish oil resulted in a surplus value with respect to a broad spectrum of parameters characterising cachexia. The group in which all nutritional components were combined (TB+fo+hpr+leu mice) was the only group that showed significant differences *vs* TB on all read-out parameters of cachexia (e.g., weights of body, carcass, muscles and fat (see Table 1)). The suggested additive effects of single nutritional components to the total combination are best illustrated by the data on tibialis muscle mass (mTA, Figure 1). These data clearly indicate a surplus value of a multi-nutritional component approach. We suggest that the observed additive effects originate from presumed differences in mechanistic targets of these components, that is, (1) stimulation of anabolic signals by supplementation of building blocks (essential amino acids) and by stimulation of mTOR (leucine), (2) reduction of protein catabolism by the reduction of inflammatory and hormonal responses (fish oil) and downregulation of the signalling pathway leading to protein breakdown (leucine), and the possible interaction(s) between these mechanisms. The second experiment confirmed the efficacy of the nutritional combination on body composition maintenance. Moreover, in this experiment the combination of ingredients also improved parameters reflecting physical performance like muscle function and daily activity patterns.

Organ (wet) mass of kidney, liver, intestine, thymus and heart were unaffected or decreased with increased cachexia. Nutritional supplementation resulting in increased carcass weight had no effect or partly normalised the loss in organ mass. Next to that, none of the selected ingredients increased tumour size. The complete nutritional combination showed a reduction in tumour size in experiment B.

We found no effect of single nutrients or their combination on food intake in the present study. Clinical data indicate that BCAA supplementation reduces the incidence of anorexia in cancer and malnourished patients and in patients with liver cirrhosis (Cangiano *et al*, 1996; Hiroshige *et al*, 2001; Marchesini *et al*, 2003; Laviano *et al*, 2005, 2006; Biolo *et al*, 2006). In a placebo-controlled multi-center trial in cirrhotic patients (*n*=174), 1 year supplementation with BCAA resulted in a decreased prevalence of anorexia from 52 to 25%, whereas anorexia prevalence remained unchanged in the control group (Marchesini *et al*, 2003). The incidence of anorexia also decreased in the BCAA supplemented group, whereas it did not change in the placebo group in a study in cachectic cancer patients supplemented with 14.4 g/day BCAA (7.1 g leucine) (Cangiano *et al*, 1996). Moreover, caloric intake in the BCAA group increased. Cota *et al* (2006) found contradictory results *in vivo* and reported an anorectic effect of Leucine after central administration in healthy rats. Several explanations were given by Laviano *et al* (2006) for this discrepancy supported by results from clinical trials. First, Cota *et al* (2006) used healthy rats of normal weights, whereas Laviano *et al* (2006) performed their study in anorectic weight-losing cancer patients, aiming to stimulate muscle weight gain in these patients. Second, leucine may exert a different effect when supplemented balanced with other amino acids through the oral route compared with injection directly into the central nervous system. The fact that our data in the C26 mice model revealed no effect of leucine supplementation on food intake may be related to the absence of anorexia in our cachectic model setup.

C26-tumour inoculation induced a loss of muscle function, as reported previously (Gorselink *et al*, 2006). A large part of the reduction of muscle function was explained by a reduction in muscle mass. These findings are in accordance with clinical data. Gogos *et al* (1998) reported a significantly higher Karnofsky performance status in malnourished patients supplemented for 40 days with 18 g of n-3 PUFA (3 g EPA and 2 g DHA) compared with placebo. These data suggest that improvement of physical activity may occur even before a significant weight gain is achieved. This may indicate that for maintaining normal life activities, preventive treatment to reduce muscle wasting is recommended. In our experimental setup, all tumour-induced muscle mass-dependent decreases in muscle function could be significantly restored by supplementation with the specific nutritional combination. These data are supported by clinical trial data of Barber *et al* (2001) reporting an improved functional performance after 3 and 7 weeks of supplementation with 2.2 g EPA+0.96 g DHA in unresectable pancreatic cancer patients. The improved physical performance coincided with increased BW and appetite. Next to muscle mass-dependent changes in muscle function, as described by Weber *et al* (2008), also muscle mass-independent loss of function is suggested by presented data (Figures 2D and 3D). A tumour-related, muscle mass-independent decrease in muscle function has not been described before. Weber *et al* (2008) described in humans that cachexia is associated with a loss of muscle volume, but not of functionality. It might be that the technique used in this study is more accurate because muscles can be weighed instead of being estimated, which might result in less variation in the results. Because the largest part of function loss is in muscle mass dependent, the muscle mass independent changes might get lost in the noise of variation of the data. This compromised muscle function became especially manifest in the maximal contraction velocity after exercise of moderate strength (Figure 3C). The muscle mass-independent decrease in muscle function could also be partly restored by supplementation with the specific nutritional combination (TB-SNC). These results indicate that the nutritional combination restores both muscle mass-dependent and muscle mass-independent decreases in muscle function.

Asthenia, resulting from cancer cachexia, leads to a reduced daily activity. Indeed, in the presence of a tumour, daily activity levels of mice decreased over time which is in line with clinical reports of cancer patients (Mormont and Levi, 1997; Mormont *et al*, 2000). It is not clear what mechanism induces the reduction in activity in cachectic cancer patients. Reduced muscle mass and decreased muscle force may contribute to the deterioration in activity. In addition, the tumour-induced inflammatory response might further reduce the daily activity. Physical activity is a major determinant of quality of life (Moses *et al*, 2004). The complete nutritional combination tested maintained activity compared with TB-CON mice. This effect may be directly related to the better maintained physical performance (improved muscle mass and function). The influence of the nutritional combination on other factors involved in physical performance, however, would need further examination.

Chevalier *et al* (2003) reported that patients with advanced colorectal cancer showed less contrast between day time and night time activity (nocturnal sleep). Individual activity patterns have even been suggested to be predictive of the patients’ survival, tumour response and quality of life (Mormont and Levi, 1997; Mormont *et al*, 2000). The possibility of a tumour-induced disturbance in diurnal activity patterns is supported by our data, indicating a tumour-related shift in activity from the dark to the light period. The specific nutritional combination tested shows a clear trend to reduce this effect. Normal sleep patterns are critically dependent on the circadian release of melatonin from the pineal gland. DHA-enriched formulas have been reported to normalise melatonin secretion in (*n*-3)-deficient rats (Zaouali-Ajina *et al*, 1999), this might also be an explanation for the results obtained in our experiments.

Further studies in patients are needed to confirm the anti-cachectic properties of a nutritional supplement containing at least leucine-enriched high protein and fish oil. Also the mechanistic effects of the nutritional combination on inflammatory processes warrant further research. When anorexia is present in this patient group, the use of a high caloric supplement seems valid. In such a setup, the effect of BCAAs in the nutritional combination on both anorexia and cachectic parameters could be evaluated. Moreover, based on the results from this study it is clear that more attention should be paid to prevention of cachexia to maintain quality of life for the patient.

In conclusion, this specific nutritional combination of high protein, leucine and fish oil improved the cachectic outcome of mice inoculated with the C26 adenocarcinoma cell line. The carcass, fat and muscle mass increased and the muscle function and daily activity improved when compared with tumour-bearing mice on the control diet. These data show that single ingredient interventions have limited value, and support the need for a balanced combination of different ingredients to enable a multi-targeted intervention to achieve beneficial effects in the complex conditions of cancer cachexia, as reflected in changes of body composition, muscle functionality and daily activity. The findings are clinically highly relevant, because muscle function and daily activity contribute to a great extent to the quality of life of the cancer patient (Morrow *et al*, 2002). In addition, an improved condition of a patient, as reflected in body composition and physical performance, contribute to compliance of the patient to the anti-cancer therapy (Andreyev *et al*, 1998).

## Figures and Tables

**Table 1:** Effects of single or combined nutritional components on body composition, and food intake (experiment A)

**(A) Carcass, tumour and fat masses at section (grams at day 20)**							
**Treatment**	***N***	**Carcass weight**	***P***	**Body weight**	***P***	**Tumour weight**	***P***
Con	40	24.4±0.3	0.000^*^	24.4±0.3	0.001^*^	0.0±0.0	0.0000^*^
TB-Con	40	20.7±0.4	—	22.8±0.4	—	2.2±0.1	—
TB-hpr+leu	10	20.0±0.6	0.807	21.8±0.6	0.992	1.8±0.1	0.1472
TB+fo	10	20.9±0.8	0.226	23.0±0.8	0.238	2.1±0.1	0.6854
TB+fo+hpr	10	22.2±0.8	0.034	24.2±0.7	0.038	2.0±0.1	0.5053
TB+fo+hpr+leu	22	22.7±0.6	0.010^*^	24.4±0.5	0.019^*^	1.7±0.1	0.0659
							
**(B) Food intake (gram per day)**							
**Treatment**	***N***	**Day 1**	**Day 7**	**Day 14**	**Day 17**	**Day 19**	**Day 20**
Con	40	4.5	3.8	4.0	3.8	3.7	3.6^*^
TB-con	40	4.2	3.9	3.8	3.8	3.5	2.9
TB-hpr+leu	10	4.7	3.8	4.0	3.1	3.7	2.9
TB+fo	10	5.4	3.9	4.1	4.0	3.1	2.3
TB+fo+hpr	10	4.4	3.9	3.9	3.7	3.3	2.6
TB+fo+hpr+leu	22	4.4	3.5	4.1	3.5	3.4	3.0

Con=mice receiving control diet A (AIN93), TB-con=tumour-bearing mice receiving control diet A (AIN93), hpr=high protein, leu=leucine, fo=fish oil.

Data as means±s.e.m.: ^*^significantly different from TB-con (*P*<0.02, *k*=5, *α*=10%) (For more details about statistics, see the Materials and Methods section).

**Table 2:** Effects of the specific nutritional combination (SNC) on cachexia parameters (experiment B)

**(A) Body, tumour and carcass weight at section (g at day 20)**						
**Treatment**	***N***	**BW**	**Delta BW**	**TW**	**CW**	**Delta CW**
CON	10	28.0±0.7^*^	5.3±0.5^*^	0.0±0.0^*^	28.0±0.7^*^	5.3±0.5^*^
TB-CON	17	20.8±0.5	−0.7±0.4	2.1±0.1	18.7±0.4	−2.8±0.4
TB-SNC	18	23.1±0.6^*^	0.9±0.6^*^	1.7±0.1^*^	21.4±0.6^*^	−0.7±0.7^*^
						
**(B) Organ weights at section (mg at day 20)**						
**Treatment**	***N***	**Epididymal fat**	**mTA**	**mG**	**mEDL**	**mS**
CON	10	443±37^*^	44.5±1.3^*^	141±4^*^	9.2±1.1	6.7±0.5^*^
TB-CON	17	87±18	33.4±0.9	108±2	7.8±0.2	5.3±0.2
TB-SNC	18	189±20^*^	38.1±0.9^*^	118±3^*^	8.3±0.5	5.7±0.2^*^
						
**(C) Change in BW in time**						
		**Change in BW (change in g: days 0–20 when compared to day 1)**				
**Treatment**	***N***	**0**	**6**	**10**	**15**	**20**
CON	10	1.0±0.3	3.6±0.4	4.3±0.7	5.5±1.0^*^	6.3±1.4^#^
TB-CON	17	0.6±0.3	2.7±0.3	3.3±0.3	3.7±0.4	−0.1±0.5
TB-SNC	18	0.9±0.2	3.4±0.3	4.4±0.4	4.6±0.4	1.8±0.6^#^
						
**(D) Food intake in time**						
			**Food intake per day (g)**			
**Treatment**	***N***		**8**	**13**	**17**	**19**
CON	10		4.4±0.2	4.3±0.2	4.4±0.3	3.5±0.6
TB-CON	17		4.0±0.2	4.1±0.1	3.7±0.3	3.5±0.5
TB-SNC	18		4.2±0.1	4.5±0.2	4.8±0.3	4.4±0.4

CON=mice receiving control diet B, TB-CON=tumour-bearing mice receiving control diet B, TB-SNC=tumour-bearing mice receiving the specific nutritional combination. BW=body weight; CW=carcass weight; delta BW=BW day 20 minus BW day 0, delta CW=CW day 20 minus CW day 0, TW=tumour weight, mTA=muscle Tibialis Anterior, mG=muscle Gastrocnemius, mEDL=muscle Extensor Digitorum Longus, mS=muscle Soleus.

Data as means±s.e.m.; ^*^significantly different from TB-CON (*P*<0.05, *k*=2, *α*=10%) ^#^significantly different from TB-CON for the whole curve (*P*<0.05, *k*=2, *α*=10%) (For more details about statistics, see the Materials and Methods section).

**Table 3:** Effects of the specific nutritional combination (SNC) on cancer cachexia-induced organ mass changes (experiment B)

**Organ masses at section (grams at day 20)**							
**Treatment**	***N***	**Kidney**	***P***	**Liver**	***P***	**Thymus**	***P***
CON	10	422±10.9	0.000^*^	1196.8±123.3	0.002^*^	40.3±10.9	0.0000^*^
TB-CON	17	342±8.0	—	1050.1±92.3		18.5±6.9	
TB-SNC	18	385±9.3	0.001^*^	1101.1±125.5	0.191	22.3±7.3	0.01749
							
**Treatment**	***N***	**Heart**	***P***	**Lung**	***P***		
CON	10	150.9±3.1	0.000^*^	161.9±4.4	0.715		
TB-CON	17	129.4±2.7	—	159.3±3.7	—		
TB-SNC	18	133.6±3.1	0.290	167.0±4.8	0.196		

CON=mice receiving control diet B, TB-CON=tumour-bearing mice receiving control diet B, TB-SNC=tumour-bearing mice receiving the specific nutritional combination.

Data as means±s.e.m.; ^*^significantly different from TB-CON (*P*<0.05, *k*=2, *α*=10%) (For more details about statistics, see the Materials and Methods section).
