# Dietary supplementation with a specific combination of high protein, leucine, and fish oil improves muscle function and daily activity in tumour-bearing cachectic mice

**DOI:** 10.1038/sj.bjc.6605005

**Published:** 2009-03-31

**Authors:** K van Norren, D Kegler, J M Argilés, Y Luiking, M Gorselink, A Laviano, K Arts, J Faber, H Jansen, E M van der Beek, A van Helvoort

**Correction to**: British Journal of Cancer (2009) **99,** 713–722. doi: 10.1038/sj.bjc.6604905

During correction of the above manuscript, the second affiliation of the lead author (Klaske van Norren) was omitted. The full affiliations are now shown below.

K Van Norren^*^^,1,5^, D Kegler^1^, JM Argilés^2^, Y Luiking^1^, M Gorselink^3^, A Laviano^4^, K Arts^6^, J Faber^1^, H Jansen^1^, EM van der Beek^1^ and A van Helvoort^1^

^1^Danone Research – Centre for Specialised Nutrition (formerly known as Numico Research), Wageningen, The Netherlands; ^2^Cancer Research Group, Departament de Bioquímica i Biologia Molecular, Facultat de Biologia, Universitat de Barcelona, Spain; ^3^Center for Innovative Consumer Studies, Wageningen UR, The Netherlands; ^4^Department of Clinical Medicine, University La Sapienza, Rome, Italy; ^5^Nutrition and Pharmacology Group, Division of Human Nutrition, Wageningen University, The Netherlands; ^6^Former employee of Numico Research, Danone Research – Centre for Specialised Nutrition (formerly known as Numico Research), Wageningen, The Netherlands

